# Multifunctional nanoplatforms application in the transcatheter chemoembolization against hepatocellular carcinoma

**DOI:** 10.1186/s12951-023-01820-7

**Published:** 2023-02-27

**Authors:** Gang Yuan, Zhiyin Liu, Weiming Wang, Mengnan Liu, Yanneng Xu, Wei Hu, Yao Fan, Xun Zhang, Yong Liu, Guangyan Si

**Affiliations:** 1 Department of Intervention Radiology, Traditional Chinese Medicine Hospital Affiliated to Southwest Medical University, Luzhou, 646000 China; 2 State Key Laboratory of Quality Research in Chinese Medicine, Macau Institute for Applied Research in Medicine and Health, Macau University of Science and Technology, Taipa, Macau SAR China; 3 Department of Neurology, The Affiliated Hospital of Southwest Medical University, Luzhou, 646000 China; 4 Department of General Surgery (Vascular Surgery), The Affiliated Hospital of Southwest Medical University, Luzhou, 646000 China; 5 National Traditional Chinese Medicine Clinical Research Base and Department of Cardiovascular Medicine, The Affiliated Traditional Chinese Medicine Hospital of Southwest Medical University, Luzhou, China; 6 Department of Anus and Intestine Surgery, Traditional Chinese Medicine Hospital Affiliated to Southwest Medical University, Luzhou, 646000 China

**Keywords:** Nanoparticles, Multifunctional, Transarterial chemoembolization, Hepatocellular carcinoma

## Abstract

Hepatocellular carcinoma (HCC) has the sixth-highest new incidence and fourth-highest mortality worldwide. Transarterial chemoembolization (TACE) is one of the primary treatment strategies for unresectable HCC. However, the therapeutic effect is still unsatisfactory due to the insufficient distribution of antineoplastic drugs in tumor tissues and the worsened post-embolization tumor microenvironment (TME, e.g., hypoxia and reduced pH). Recently, using nanomaterials as a drug delivery platform for TACE therapy of HCC has been a research hotspot. With the development of nanotechnology, multifunctional nanoplatforms have been developed to embolize the tumor vasculature, creating conditions for improving the distribution and bioavailability of drugs in tumor tissues. Currently, the researchers are focusing on functionalizing nanomaterials to achieve high drug loading efficacy, thorough vascular embolization, tumor targeting, controlled sustained release of drugs, and real-time imaging in the TACE process to facilitate precise embolization and enable therapeutic procedures follow-up imaging of tumor lesions. Herein, we summarized the recent advances and applications of functionalized nanomaterials based on TACE against HCC, believing that developing these functionalized nanoplatforms may be a promising approach for improving the TACE therapeutic effect of HCC.

**Supplementary Information:**

The online version contains supplementary material available at 10.1186/s12951-023-01820-7.

## Introduction

Liver cancer generally refers to primary liver cancer as the sixth most common cancer and the fourth cause of cancer-related death globally [1]. Hepatocellular carcinoma (HCC) accounts for approximately 90% of all cases of primary liver cancer [2]. Ablation, surgical resection, or transplantation are radical treatments for HCC patients at an early stage [3]. However, HCC is asymptomatic in the early stage and is often ignored. Most HCC patients are diagnosed at intermediate and advanced stages, and hence more than 70% of HCC patients miss the best time for radical treatments [4]. Intra-arterial therapy approaches (e.g., transarterial embolization, TAE or transarterial chemoembolization, TACE) are considered as prioritized treatment options for HCC patients at an intermediate stage according to the Barcelona Clinic Liver Cancer (BCLC) staging system [5, 6]. As a palliative therapy, TACE plays a critical role in treating HCC as a stand-alone or combination therapy in any stage of HCC or as a neoadjuvant treatment before liver transplantation [7].

Although the liver has dual blood supply, 95–98% of the blood supply of most malignant hepatic tumors is provided by the hepatic artery, while more than 75% of the blood supply of healthy hepatic parenchyma is provided by the portal vein, which is the theoretical basis for TACE treatment of HCC [8, 9]. The basic principle of TACE is to block the blood supply of solid tumors through the hepatic arterial route and, simultaneously, administrate chemotherapeutic drugs to achieve the effects of localized embolization (ischemic effect) and chemotherapy (cytotoxic chemotherapeutic effect) [10]. In general, conventional TACE (c-TACE) uses lipiodol-based emulsion as an antineoplastic-drug carrier plus an embolizing agent to embolize the tumor-supplying artery, which enables local delivery of antineoplastic drugs by intra-arterial approach and reduces systemic side effects compared with systemic chemotherapy [11]. Nevertheless, the lack of effective drug carriers and the inability to control drug delivery to the targeted tumor tissues leads to insufficient drug distribution in tumors and rapid drug release. Therefore, HCC patients who have undergone c-TACE therapy may still suffer recurrence and some systemic adverse reactions like postembolization syndrome, including abdominal pain, fever, nausea, vomiting, diarrhea, et al. [12–14]. In addition, the hypoxic tumor microenvironment (TME) after embolization can induce the survival of viable tumor cell population and tumor neoangiogenesis, which is closely related to the progression and metastasis of solid tumors [15, 16].

Drug-eluting beads (DEBs), which were first commercially available in 2004, were invited to improve the efficacy of TACE and reduce systemic side effects [17]. The application of DEB-TACE has achieved embolization of tumor vasculature, drug delivery, and controlled release of drugs, resulting in higher concentration of drugs within the target tumor and lower systemic concentrations compared with c-TACE. Several clinical trials compared the safety and efficacy of DEB-TACE with c-TACE, indicating that DEB-TACE reduced the incidence of specific toxicity associated with doxorubicin (DOX) [18], relieved postembolization pain in patients [19], and decreased the frequency of abdominal pain and fever [20]. Nonetheless, unexpectedly, DEB-TACE carries a higher risk of hepatic artery and biliary damage than c-TACE [21, 22]. Moreover, although drug-eluting microspheres have been developed for decades, the mechanism of drug incorporation limits the drugs that may be used [23]. Generally, microspheres use either an ion-exchange method or a swelling process followed by the interaction of the drug with ionized side chains (i.e., carboxylate or sulfonate groups) to achieve drug loading [24, 25]. Therefore, only positively charged drugs with low molecular weight such as DOX, Idarubicin, and Irinotecan may be incorporated [14]. Furthermore, although a range of existing microspheres cover 30–900 μm in size [14], they still cannot reach the arterial-capillary level, reducing penetration and the total tumor volume exposed to drugs [23]. An abundance of evidence has revealed that DEB-TACE does not appear to show more efficacy advantage over c-TACE therapy, except for improving patient tolerability and reducing systemic drug reactions [19, 26, 27]. Therefore, it is urgent to develop new chemoembolization systems to improve the therapeutic effect of TACE and reduce the side effects.

Recently, the application of nanomaterials in HCC diagnosis and treatment has attracted extensive attention [28–30]. Nanomedicine has provided novel possibilities to conquer current challenges in TACE therapy. Due to the unique features of nanomaterials, such as nanoscale carriers, high specific surface area, and specific physicochemical properties, they have excellent application in drug delivery [31], specific targeting [32], enhancement of pharmaceutical properties [33], co-delivery of multiple drugs [34], and visualization of sites of drug delivery by combination with imaging modalities [35, 36]. Moreover, some nanomaterials themselves also possess therapeutic properties [37, 38]. Hence nanomaterials can provide bright prospect for early diagnosis and precise treatment of cancer [39–41]. Currently, nanoplatforms based on TACE therapy have been widely developed to enhance the therapeutic effect and the safety of treatment for HCC. The most significant superiority of multifunctional nanoplatforms in the combination of TACE therapy against HCC is that they can not only improve the distribution of chemotherapy drugs in local tumor tissue but also avoid the severe side effects caused by systemic drug administration. Compared with traditional methods, functionalized nanoplatforms also have the advantages of active targeting ability, specificity, synergy, and integration of diagnosis and treatment. In addition, several functionalized nanoplatforms have been developed to cope with the worsened post-embolization TME (e.g., hypoxia, reduced pH, angiogenesis), such as calcium carbonate encapsulated alginate microspheres that can neutralize the pH of the tumor [42], HIF-2α-targeted interventional chemoembolization multifunctional microspheres [43], Tirapazamine-loaded CalliSpheres microspheres (CSMTPZs) that can enhance synergy between TPZ and embolization against liver cancer [44], and multifunctional embolic microspheres with inhibitory effect on angiogenesis [35], which have shown great potential in enhancing chemoembolization. The preliminary results showed that these functionalized nanoplatforms can be combined with TACE to play a synergistic anti-cancer role, which shows a great prospect in enhancing the efficacy of TACE and improving the TME.

**Fig. 1:**
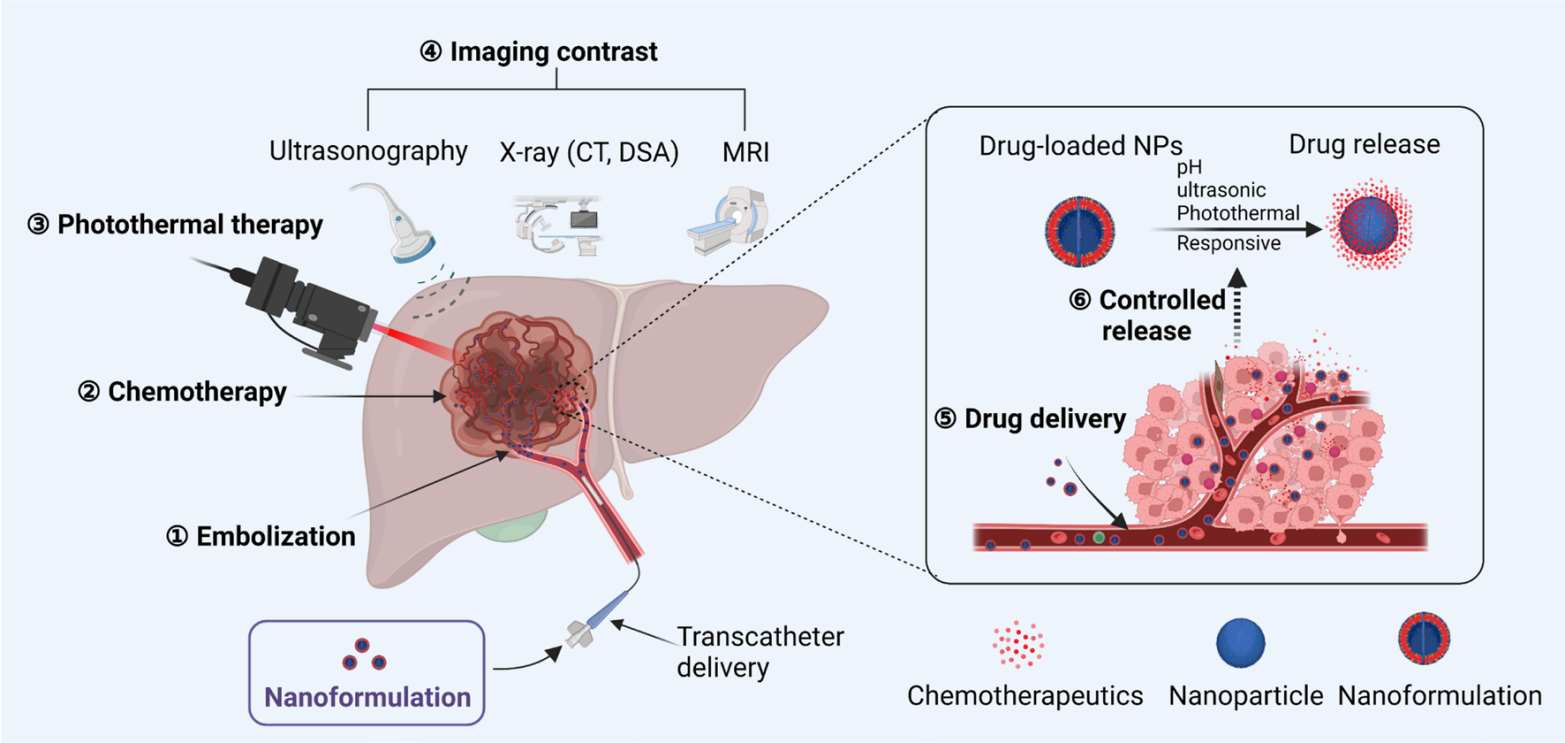
Applications of multifunctional nanoplatforms in the TACE treatment against HCC. Nanoformulations perform functions such as embolization, chemotherapy, photothermal therapy, imaging, drug delivery, and drug-controlled release. The figure has been created using Biorender

This review summarized the applications of multifunctional nanoplatforms in the TACE treatment against HCC (Fig. 1) and the related limitations and future development prospect based on the growing literature in recent years, aiming to facilitate the upgraded development of functional nanoplatforms, broaden the prospects and applications of TACE, and benefit HCC therapy.

## Rational design of multifunctional nanoplatforms for TACE procedure

The development of nanomaterials provides new possibilities to overcome the current challenges in TACE therapy for HCC, which is mainly attributed to the unique characteristics of nanomaterials in drug delivery, specific targeting, enhancement of drug properties, combined delivery of multiple drugs, and visualization of drug delivery sites by combined imaging methods [37]. As shown in Fig. 1, combining a multifunctional nanoplatform and catheter embolization in the treatment of HCC has demonstrated a powerful effect. Apart from the most basic chemotherapy and embolization function, the multifunctional nanoplatform can also realize the function of local hyperthermia and image follow-up. In addition, it acts as a carrier for drug delivery and can achieve controlled release of drugs in response to physical or chemical stimuli (such as pH, ultrasonic, or temperature changes) inside and outside the tumor. Therefore, the combination of multifunctional nanoplatforms and transcatheter embolization has apparent superiority in improving the efficacy and safety of TACE therapy for HCC and imaging follow-up after treatment. It is a prevalent strategy that various functional nanomaterials are integrated into the composites, which, as a multifunctional nanoplatform, can be applied in the TACE procedure to improve the diagnosis and prognosis of HCC.

Various nanoscale drug delivery systems have been developed, including micelles, liposomes, polymeric nanoparticles, mesoporous silica nanoparticles (MSNs), etc. [45, 46]. Herein, we mainly focused on designing the nanoplatforms based on material category for developing ideal co-delivery nanotherapeutics (Table 1), characterized by reduced systemic side effects, prolonged circulation, targeting delivery, on-demand release, reversed resistance, tumor visualization, and synergistic treatment. Obviously, the trials of these different categories of nanomaterials in the treatment of HCC via the TACE therapy provide strong support for the development of new functional nanoplatforms.

**Table 1:** List of representative nanocarrier platforms for TACE therapy of HCC

Material category	Examples of nanoplatform	Applications	Material advantage	Refs.
Liposome	PEGy-lated liposomal; nano flexible liposomes; RLBNs	Drug-carrier; embolic agent	Decrease in systemic toxicity of free drug; improve delivery to tumor tissue; prolonging drug circulation and delaying its release	[47–49]
SPION	SPIO; USPIO; SPION@PDA	MRI contrast; drug-carrier; embolic agent; PTT	Improve the detection of viable tumors; improve drug loading capacity; reduce systemic toxicity; sustained drug release; synergistic enhancement of PTT and chemotherapy; prevent drug resistance	[50–54]
Metal oxide NPs	Iron Oxide; ZrO_2_; Fe_3_O_4_; As_2_O_3;_ ATONP	MRI contrast; drug-carrier; embolic agent	Improve the detection of viable tumors; improve drug delivery and enhanced its antitumor efficacy; sustained drug release; ferroptosis amplify the antitumor efficacy	[55–61]
Metal NPs	Tantalum NPs; Gold nanospheres	Drug-carrier; X-ray contrast; embolic agents; PTT	Real-time imaging; long-term non-invasive re-examination; synergistic enhancement of PTT and TACE therapy	[62, 63]
HAP	HAP; poly-L-lysine-modified HAP nanoplex	Embolic agent; gene vector	Combined gene therapy, embolic therapy, and nanotherapy at the tumor site; prevent resistance of chemotherapies	[64, 65]
Polymeric NPs	PLGA	Drug-carrier; embolic agent	Biodegradable; sustained drug release; stabilized lipiodol emulsion	[66, 67]
Polymer gel	SELPs; hydrogels	Liquid embolic agent; X-ray contrast agent; drug-carrier	Enhanced embolization capacity; facilitate permeation of tumor vasculature; pH response; resist revascularization; imaging; prolong drug retention	[23, 68–70]

*MSN* mesoporous silica nanoparticles, *PEG* polyethylene glycol, *RLBNs* reversed lipidbased nanoparticles, *SPION* superparamagnetic iron oxide nanoparticles, *MHT* magnetic hyperthermal, *ATONP* arsenic trioxide nanoparticle prodrug, *PLGA* polylactic-co-glycolic acid, *HAP* hydroxyapatite nanoparticles, *SELPs* silk-elastinlike protein polymers

Ideal nano-carrier designed for TACE should possess high load capacity to chemotherapeutic drugs, selectivity toward cancer cells, drug-controlled release, and intra- and post-operative visualization in real-time. They can improve the solubility and chemical stability of small molecules of drugs. In addition, targeted antitumor drugs can reduce drug resistance of tumors. Moreover, controlled release of drug-loaded NPs can protect drugs from rapid excretion and biodegradation, thus improving the pharmacokinetic parameters of drugs in vivo, promoting the enrichment of drugs in local tumor tissues, and improving the anticancer-targeting ability of drugs. Therefore, developing multifunctional nanocomposites with high drug loading efficacy, targeted delivery, sustained release, and imaging properties for TACE therapy of HCC is becoming a hot research topic in recent years [45, 46, 71, 72]. As shown in Table 2, apart from drug delivery, different kinds of nano-formulations possess diverse functions such as hyperthermia, embolization, imaging, etc., which are conducive to synergistic diagnosis or monitoring, and treatment of HCC.

**Table 2:** Multifunctional nano-formulations used in TACE therapy of HCC

Nano-formulations	Materials	Applications	Drugs	Refs.
Gold nanospheres	HAuNS, lipiodol	Drug-carrier; PTT; embolic agent	DOX	[63]
Nano-clusters	pMNCs, lipiodol	Drug-carrier; embolic agent; MRI	DOX	[73]
Nanocomposites	α-Fe_2_O_3_ nanocubes, PDA, and lipiodol	Drug-carrier; PTT; embolic agent; MRI	DOX	[74]
	UiO-66/Bi_2_S_3_ NPs	Drug-carrier; PTT; embolic agent	DOX	[75]
	BSA, CuS NPs	Drug-carrier; PTT; embolic agent; MRI	DOX	[76]
Microspheres complex	Carrageenan, inhexol and SPION	Biocompatibility, Drug-carrier; embolic agent; imaging (MRI and X-ray)	DOX	[53]
	Gelatin and Fe_3_O_4_ NPs	Drug-carrier; PTT; embolic agent; MRI	ADR	[60]
	Alginate and USPIO NPs	Biocompatibility; Drug-carrier; embolic agent; MRI	AMO	[52]
	Calcium alginate and tantalum NPs	Drug-carrier; embolic agent; X-ray imaging real-time	DOX	[62]
	USPIO nanocluster and alginate	Drug-carrier; embolic agent; MRI	MEAN	[77]
	PDA, SPION	Drug-carrier; PTT; embolic agent; MRI	DOX	[54]
Microbubble complex	Albumin NPs and Lipiodol	Drug-carrier; drug release triggered by ultrasound; real-time monitoring	DOX	[78]
Hybrid composites	PVA and iron oxide nanoshell	Drug-carrier; embolic agent; MRI	DOX	[55]

*HAuNS* hollow gold nanoshells, *pMNCs* porous magnetic nano-clusters, *PDA* polydopamine, *CuS* copper sulfide, *SPION* superparamagnetic iron oxide nanoparticles, *USPIO* ultrasmall superparamagnetic iron oxide, *MEAN* 6-methoxyethylamino numonafide, *PVA* poly vinyl alcohol, *ADR* Adriamycin, *AMO* amonafide

## Strategies for diagnosis and TACE treatment of HCC based on multifunctional nanomaterials

According to the current literature about the application of nanomaterials in the diagnosis and TACE treatment of HCC, several common strategies are summarized as follows.

### Visualization (imaging)

Recently, the use of the contrast agent effect of nanomaterials combined with chemoembolization in treating HCC has become a hot topic. Although microspheres are widely used in the TACE procedure [14], they must be mixed with contrast agents (e.g., iodine contrast medium) to achieve visualization during the therapeutic process since the microspheres cannot be visualized under the X-ray, which to some extent affects the accuracy of embolization and limits postoperative tracking and evaluation. Therefore, developing a visualized embolization system is highly important in interventional therapy [62, 79, 80]. As shown in Table 3, we listed the representative multifunctional nano-embolization system for imaging in TACE or TAE therapy of HCC.

**Table 3:** List of nano-embolization system for imaging in TACE or TAE therapy of HCC

Imaging	Embolism system	Contrast agent	Drugs	Synergistic therapy	Refs.
Ultrasonic imaging	DOX-NPs-MB	Microbubbles	DOX	Ultrasound and TACE therapy	[78]
X-ray imaging(CT/DSA)	Fe@EGaIn/CA	Fe@EGaIn	DOX	PTT, PDT and TACE therapy	[36]
	BaSO_4_/ALG	BaSO_4_	N/A	TAE therapy	[81]
	Ta@CaAlg	Tantalum NPs	DOX	TACE therapy	[62]
	MSMC	MoS_2_ nanosheets	N/A	TAE and microwave ablation therapy	[82]
MRI	Fe@EGaIn/CA	Fe@EGaIn NPs	DOX	PTT, PDT and TACE therapy	[36]
	SPION@PDA	SPION	DOX	PTT and TACE therapy	[54]
	Fe_3_O_4_-MS	Fe_3_O_4_ NPs	ADM	TACE, microwave ablation, and ferroptosis	[60]

*DOX-NPs-MB* doxorubicin-loaded albumin nanoparticle-conjugated microbubble, *Fe@EGaIn* eutectic gallium-indium alloy nanodroplets containing iron nanoparticles, *Fe@EGaIn/CA* calcium alginate microspheres loaded with Fe@EGaIn NPs, *BaSO4/ALG* barium alginate microspheres loaded with in situ synthesized BaSO_4_ particles, *Ta@CaAlg* calcium alginate microspheres loaded with tantalum NPs, *MSMC* *MoS*_*2*_ nanosheets encapsulated in sodium alginate microcapsules, *SPION@PDA* polydopamine coated superparamagnetic iron oxide NPs, *Fe*_*3*_*O*_*4*_*-MS* homogenous gelatin microspheres co-loaded Fe_3_O_4_ NPs, *ADM* adriamycin

For the application of nanomaterials in ultrasound imaging, NPs are usually integrated into microbubbles which are ultrasonic contrast agents. Nanoparticle-coated microbubbles are stable and echogenic, and they can release the cargo of drug-containing NPs with an ultrasound trigger [83]. Incorporating drug-loaded nanocarrier microbubbles in TACE preparation can monitor the delivery of drug to the tumor in real time and enhance the therapeutic efficacy of TACE (Additional file 1: Fig. S1) [78]. Based on the unique advantages of ultrasound and nanotechnology, ultrasound nanomedicine has been regarded as a highly promising and valuable tumor-specific theragnostic methodology, which will pave a novel and efficient way for combating cancer [84, 85].

Biodegradable and image-visible (e.g., CT or MRI) microparticles synthesized by NPs that can be tracked in vivo have been developed as a form of multifunctional nanocomposite (Table 3). Introducing radiopaque elements (e.g., I, Ag, Ba, Ta, etc.) into microspheres to enable visualization of the microspheres under X-ray is theoretically feasible. Studies have shown that DC Bead LUMI™ containing iodine [86], BaSO_4_-sodium alginate microspheres [87], tantalum NPs calcium alginate microspheres (Ta@CaAlg) [62], and molybdenum sulfide nanosheets encapsulated in sodium alginate microcapsules (MSMC) [82] can realize X-ray visualization under both DSA and CT, which can act as visualized embolization systems for TACE treatment of HCC. This is very beneficial for precise TACE treatment, as well as CT follow-up of tumor lesions after therapy.

In addition, MR visualization microspheres are usually prepared by adding MR contrast agents to the microspheres, including paramagnetic lanthanides, such as cerium, lanthanum, rubidium, praseodymium, gadolinium [88, 89]. Clinically, gadolinium-based chelates are the mainstay of contrast agents for MRI. Nevertheless, Food and Drug Administration (FDA) has issued many warnings about their potential retention in patients’ bodies, raising safety concerns because their toxicity can elicit severe side effects [90]. Fortunately, the nontoxic and biodegradable natural NPs such as iron oxide nanoparticles (IONPs) are potentially attractive alternatives [91]. Superparamagnetic iron oxide (SPIO) MR contrast agents such as ferumoxides and ferucarbotran are composed of nano-sized iron oxide crystals coated with dextran or carboxydextran, which are clinically used for liver MRI [92]. As a potentially helpful theranostic agent, a hybrid composite made up of SPIO nanoshells encapsulating the anticancer drug DOX and bound together by polyvinyl alcohol (PVA) has been developed to treat HCC with TACE approach [55, 93]. This treatment strategy not only achieves the functions of chemotherapy and embolization for tumors but also uses the MR response of SPIO nanomaterials, which is conducive to postoperative MRI follow-up of lesions. Recently, studies reported that SPIO nanoclusters alginate microspheres [52], temperature-sensitive lipoid-barium alginate microspheres (Tsl-BA-MS) [94], alginate-USPIO microspheres composed of calcium alginate-USPIO nanoclusters [79] could be visualized under MRI. However, we must be aware that these MR visualizing microspheres can only be used for the follow-up of tumor lesions after TACE and they cannot realize real-time visualization during the TACE process. Therefore, there is a need to develop embolic materials that can be visualized in real-time during the operation to achieve more precise and controlled intervention.

### Targetability

#### Active targeting

**Fig. 2:**
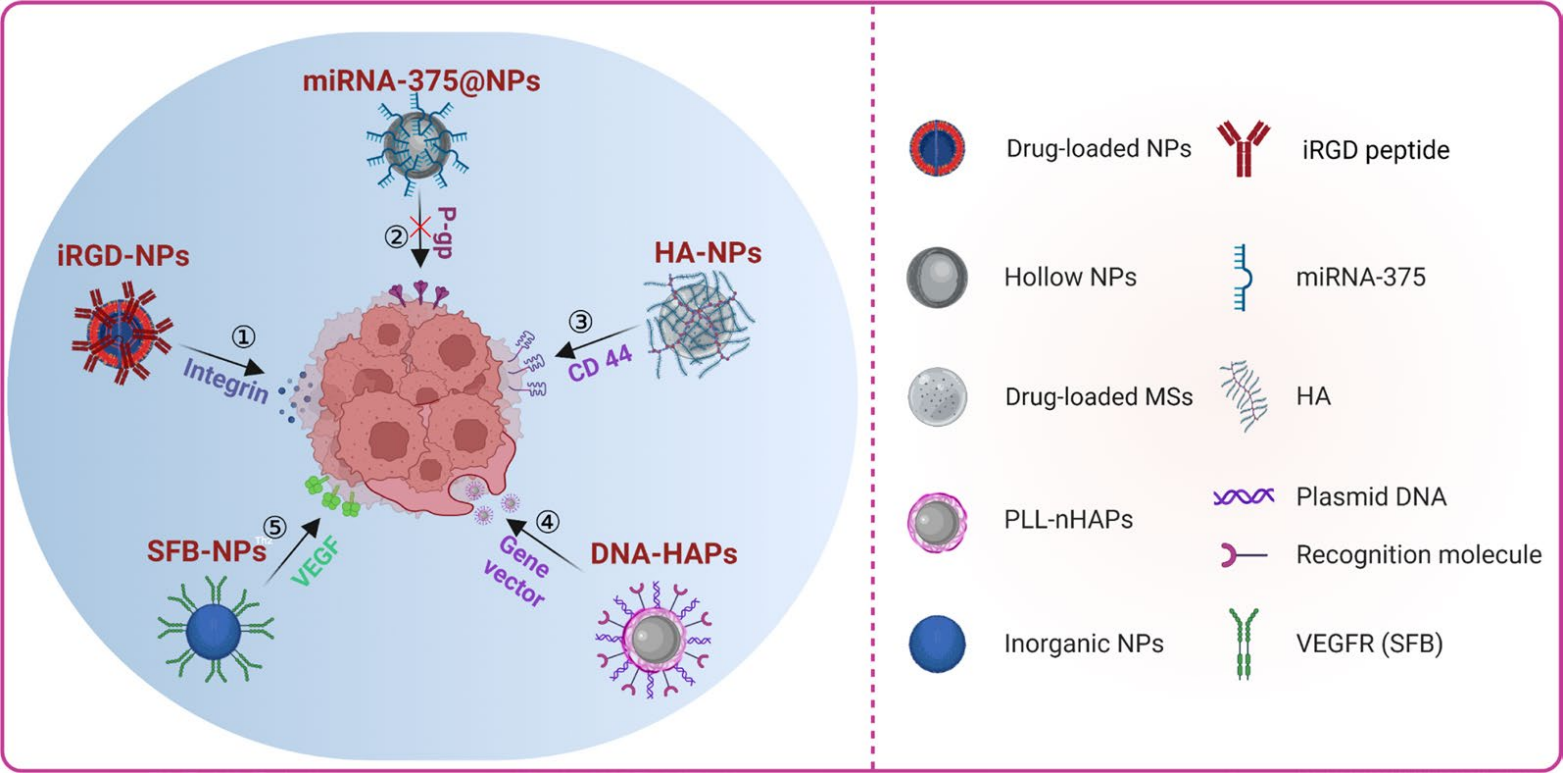
Application examples of active targeting strategy based on nanocarriers in TACE treatment of HCC. ① iRGD-modified nano-formulation target integrin improves the tissue distribution of the drug and its antitumor efficacy [57]. ② Delivery of miR-375 by hollow NPs inhibits P-glycoprotein (P-gp) expression and overcomes multiple drug resistance in HCC [95]. ③ Hyaluronic acid (HA)-based nanocarriers improve the cellular internalization of chemotherapeutics and anticancer activities via targeting CD44 receptor-mediated endocytosis [96]. ④ Hydroxyapatite NPs (nHAP) serve as gene vectors to elevate and synergize the therapeutic efficacy of TAE and target gene therapy [64]. ⑤ Application of TACE combined with targeted NPs delivery of sorafenib (SFB-NPs) system recognizes and antagonizes VEGF specifically in treating HCC with microvascular invasion [97]. The figure has been created using Biorender

The targetability of nanocarrier is crucial to improve treatment efficacy and reduce side effects during TACE treatment for HCC. Drug-loaded NPs (nanomedicines) can be delivered into tumor tissues through active or passive targeting. Active targeting is mainly based on specific expression of receptors or proteins on tumor target cells. Nanocarriers are designed to precisely recognize these receptors or proteins ligands through “receptor-ligand” binding mode to achieve the purpose of active targeting. Due to the active targeting properties of nanocarriers, some specific ligands expressed on tumor surfaces, including peptides [57], proteins [95], antibodies [96], and oligonucleotides [64], can become targets of the nanocarrier delivery systems for cancer treatment (Fig. 2).

### *Integrins*

The recurrence and metastasis of HCC after TACE therapy remain the leading cause of poor prognosis of HCC. Tumor metastasis is a complex process involving multiple factors, among which the integrin-mediated adhesion of tumor cells to the basement membrane is one of the critical factors leading to tumor cell infiltration and metastasis [98]. Integrins are receptor molecules on the cell surface that mediate the adhesion between cells or cells and extracellular matrix (ECM) [99]. Therefore, integrins can be used as therapeutic targets to prevent liver cancer recurrence and metastasis. Studies showed that transarterial administration of GRGDSP (Gly-Arg-Gly-Asp-Ser-Pro, integrin-inhibitor) loaded nanocarriers combined with TACE significantly delayed tumor growth and intrahepatic metastases compared with TACE alone or TACE plus GRGDSP in an animal model of HCC [98, 100]. Another study evaluated the effect of transarterial infusion of iRGD-modified and doxorubicin-loaded zirconia-composite nanoparticles (R-DZCNs, which can selectively recognize and bind to integrin) with lipiodol on improving the distribution of DOX in liver tumors and its antitumor efficacy. The results suggested that transarterial infusion of R-DZCNs with lipiodol improved the distribution of DOX and enhanced its antitumor efficacy [57]. The successful application of these strategies in animal experiments provides new reference for reducing the recurrence and metastasis after interventional embolization therapy of HCC.

### *P-glycoprotein*

Drug resistance, which is regulated by P-glycoprotein (P-gp), is a critical obstacle to cancer treatment [101]. P-gp is located on cell membrane and is a typical molecular pump that protects cells from invading harmful foreign molecules. It can shield or pump harmful components, such as chemotherapy drugs, out of the cell, resulting in drug resistance. P-gp constantly searches for foreign hydrophobic molecules and is described as a “security guard” protecting cell safety. Therefore, P-gp is an essential target for treating tumor drug resistance.

Studies have shown that miR-375 can inhibit the expression of P-gp by inhibiting the expression of hepatoma astrocyte up-regulated gene-1 (AEG-1) and induce apoptosis in hepatoma cells [102]. Based on this finding, researchers designed an experiment on lipid-encapsulated hollow mesoporous silica NPs (HMSN) carrying miR-375 and DOX (LHD/miR-375) in the treatment of multidrug resistance in HCC [95]. The results showed that LHD/miR-375 overcame drug efflux and imported miR-375 and DOX into multidrug-resistant hepatoma cells or tumor tissues, and that miR-375 carried by LHD/miR-375 inhibited the expression of P-gp in HCC cells after it was ingested through phagocytosis and endocytosis mediated by reticular and fossa proteins and then released from the advanced endosome (Additional file 1: Fig. S2). The synergistic effect of miR-375 and HMSN significantly increased the uptake of DOX by HCC cells and inhibited the growth of HCC cells. In addition, studies also showed that miR-375 and DOX played a synergistic antitumor effect by promoting apoptosis [95]. Therefore, the combination of a nano-drug delivery system and P-gp inhibitor may have potential application value for MDR treatment of HCC. On this basis, developing a drug-delivery nanoplatform for TACE therapy of HCC may have further clinical application value.

### *CD44*

CD44 is a multifunctional transmembrane glycoprotein overexpressed in numerous cancer cells’ cell membrane. It is also a receptor of hyaluronic acid (HA), mainly involved in cell proliferation, migration, and angiogenesis, and plays an essential role in cancer metastasis process [103, 104]. HA is a naturally occurring polysaccharide that is biocompatible, biodegradable, and non-antigenic, and is widely used in biomedical applications as a conjugate, hydrogel, nanogel, and many other roles [105]. Recently, HA has been developed into nanoplatforms that recognize and bind CD44 for cancer therapy [103, 104, 106].

There is increasing evidence proving that HA-based nanocarrier systems targeting CD44 receptors play a positive role in TACE therapy for HCC. Lee et al. developed DOX-loaded, hyaluronic acid-ceramide (HACE) nanoassembly-releasing PLGA microspheres for TACE therapy of liver cancer [96]. The result suggested that these microspheres accelerated cellular accumulation of DOX and enhanced its anticancer activities. In addition, HA-based nano-sized drug carrier-containing Gellan gum microspheres were also developed as a multifunctional embolic agent for TACE therapy [107]. The microspheres can effectively improve the stability of anticarcinogen, increasing drug-targeted affinity towards cancer cells and drug availability. Undoubtedly, the application of CD44 as a target, based on the HA-nano drug delivery system in treating HCC by TACE, has become a new therapy model, which will provide new therapeutic strategies for targeted therapy of medium and advanced HCC.

### *Gene target*

Gene therapy has become a promising new treatment strategy for unresectable HCC as hepatocellular carcinogenesis correlates with the activation of oncogenes and dysfunction of anti-oncogenes [108]. Designing a safe and effective transfection vector is the key to gene therapy. Although viral vectors are currently considered to be most effective, safety concerns have limited their clinical application, making non-viral vectors the focus of attention of clinicians and researchers [109, 110].

Hydroxyapatite nanoparticles (nHAP) as a gene vector transarterial embolization (TAE) can block tumor blood supply and deliver the agents to the tumor area; higher concentrations of drugs in tumor tissue can enhance tumor cells' specific gene uptake, making efficient, locally targeted gene therapy possible [64]. Li et al. have found that nHAP exhibit good liver cell compatibility, safety, and tumor-specific inhibition [111]. Besides, with features of a large surface and high surface energy, nano-sized nHAP can absorb DNA and form a nano polyplex [112]. A previous study explored the combined effect of p53 and Rb in local TAE gene therapy in a rabbit VX2 model of HCC. The result showed that Rb worked synergistically with p53 in combined therapy mediated by a poly-L-lysine-modified nHAP nano polyplex to augment the antitumoral effect through the downregulated expression of essential genes related to apoptosis, necrosis, growth, differentiation, and multidrug resistance of tumor cells [65]. Another study also explored the synergism of wt-p53 and synthetic material (such as poly-lysine modified nHAP) in local nano-TAE gene therapy of hepatoma [113]. These studies created a new gene delivery route using nHAP, which promoted the application of TAE-target gene therapy in HCC.

### *VEGF*

Tumor tissue hypoxia induced by TAE will lead to the expression of vascular endothelial growth factor (VEGF), thereby accelerating the formation of new blood vessels, which creates favorable conditions for tumor recurrence and metastasis and is, therefore, one of the critical factors affecting the efficacy of TACE for HCC [114, 115]. Sorafenib and other anti-angiogenic targeted drugs are based on these therapeutic targets. However, in clinical practice, oral anti-angiogenic targeted drugs may cause many adverse events such as decreased appetite, nausea, vomiting, diarrhea, fatigue, hand-foot skin reaction, hypertension, and weight loss, leading many patients to discontinue or reduce the dose, and the rebound of disease after discontinuation will lead to more serious consequences [116]. In this regard, nano-drug delivery systems have shown remarkable therapeutic advantages. For example, an in vitro study reported that biodegradable polylactic acid drug-eluting microspheres loaded with sorafenib and cisplatin had been prepared to achieve the triple therapeutic efficacy of embolization, anti-angiogenesis, and chemotherapy [117]. In addition, in some studies, sorafenib and ferric oxide NPs were co-encapsulated in poly-lactide-glycolide (PLG) microspheres, which could not only realize the angiogenesis inhibition of sorafenib but also could be used for postoperative MRI detection [35, 118]. Recently, preclinical, and clinical studies also validated the therapeutic efficacy of delivering sorafenib-eluting microspheres or nanocarrier systems used in combination with TACE for treating HCC with microvascular invasion [56, 97]. These therapeutic strategies undoubtedly provide new ideas for solving angiogenesis and preventing tumor recurrence after TACE therapy.

#### Passive targeting

**Fig. 3:**
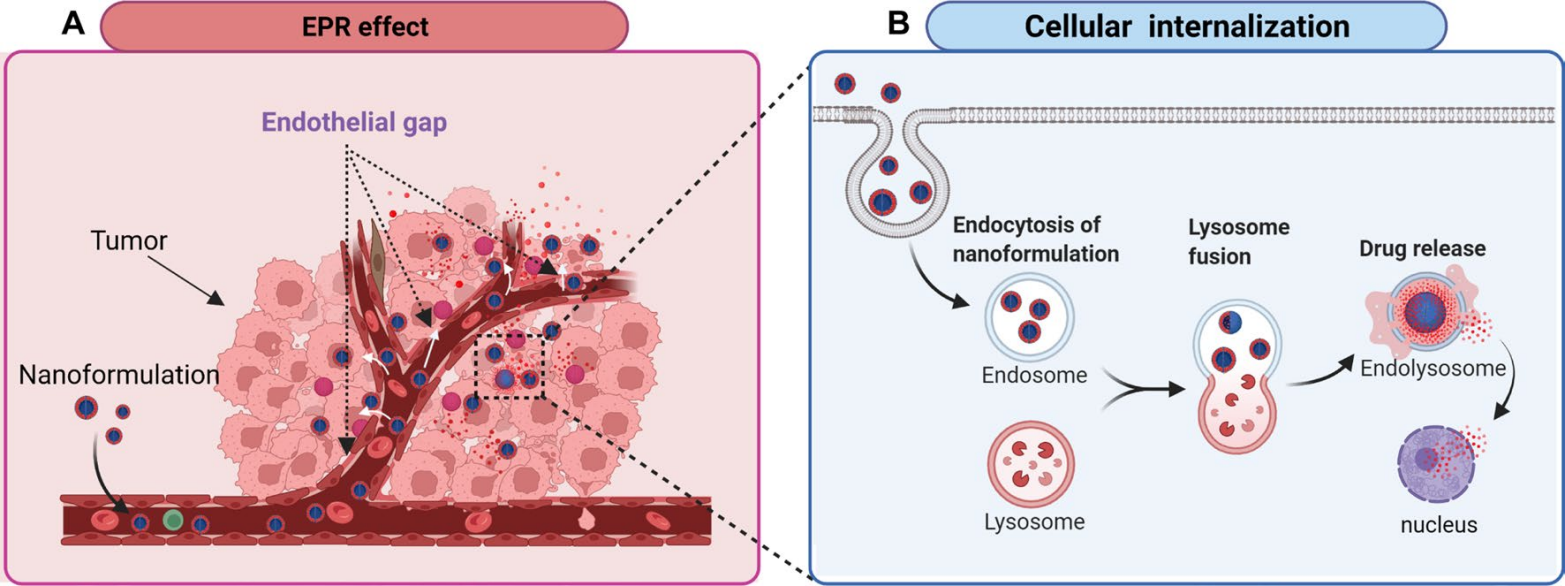
Nanocarriers can facilitate the delivery of chemotherapeutics. **A** Nanoformulations accumulation in the tumor tissue relies on the enhanced permeation and retention (EPR) effect. **B** Intracellular delivery of nanoformulations, including endocytosis, lysosomal transport, nanoparticle degradation, and drug release. The figure has been created using Biorender

Unlike the above active targeting strategies, passive targeting serves as a basis for developing macromolecular anticancer therapy. The enhanced permeability and retention (EPR) effect plays a vital role in passive targeting, which mainly relies on the anatomical and pathophysiological characteristics of solid tumors and their microenvironments, such as high vascular density in tumor tissue based on angiogenesis, intermittent tumor vasculature with leaky effects and poor drainage of tumor lymphatic system [119, 120]. As shown in Fig. 3A, due to the EPR effect, nanoformulations in circulation can enter tumor tissues through the gaps between endothelial cells in tumor blood vessels [121]. This effect further enhances the preferential accumulation of nanocarriers within tumor tissues and accelerates intracellular delivery including endocytosis, lysosomal fusion, escape, cytoplasmic release, and combining the target organelle (Fig. 3B) [46, 119, 122]. These properties of tumors provide favorable conditions for designing tumor-targeted antitumor NPs. Drug-carrying NPs can increase the accumulation of chemotherapy drugs in tumor tissues through EPR effect, improve therapeutic effects and reduce toxic and side effects by reducing unnecessary release in normal tissues [123, 124]. This unique passive targeting effect of nanomaterials on tumor tissue is highly beneficial for developing multifunctional nanoplatforms for TACE therapy of HCC, as it synergically enhances the safety of the transarterial administration and promotes more efficient drug absorption by the tumor.

### Drug delivery

#### Facilitate drug delivery capacity

Nanocarriers can facilitate drug delivery mainly by enhancing drug loading efficiency and intracellular uptake. Surface modification of nanomaterials is a common functionalization strategy through which nanomaterials are functionalized with organic molecules such as polymers [125], peptides [126], or cell membrane coated-biomimetic NPs [127] on the surfaces of these materials to facilitate drug delivery capacity in cancer theranostics. For example, to increase drug loading content, adding charged functional groups (polymeric additives: polyallylamine hydrochloride, PAH, giving them a positive charge, and polyacrylic acid, PAA, giving them a negative charge) on the surface of calcium phosphate NPs to endow them with a corresponding charge so that the target drug with the opposite charge can be loaded through efficient adsorption [128]. In terms of increasing the intracellular uptake of anticancer drugs, peptides with a particular function, like cell-penetrating peptides (CPPs), have recently emerged as efficient molecular transporters for intracellular drug delivery to conquer biological barriers and deliver cell-impermeable cargoes into cells [126]. The conjugation of CPPs to polymeric nanoplatforms enhances drug delivery efficiency, thus increasing their therapeutic efficacy. In addition, another nanoplatform, namely cell membrane coated-biomimetic NPs, can overcome biological barriers by escaping from immune clearance, opsonizing, and negotiating with the vascular system [127, 129, 130]. All these nanoplatforms have outstanding contributions towards facilitating anticancer drug delivery and are expected to play a positive role in TACE therapy of HCC.

#### Controlled drug sustained release

A critical factor limiting the therapeutic effect of c-TACE is the instability of chemotherapeutic agents in the lipiodol-TACE system. DOX, cisplatin, and 5-fluorouracil are the most commonly used chemotherapeutics for the treatment of HCC; among them, tried for TACE therapy, DOX-based chemotherapeutic agents appear to offer the highest efficacy, but with response rates of only (20 − 30) % and a minimal impact upon survival [131]. The main reason is that the dispersion of hydrophilic drugs in the hydrophobic lipiodol phase is not stable, and the drugs often separate from the emulsion, leading to an initial burst drug release [67]. The rapid release of chemotherapeutic drugs within a short period reduces the therapeutic effects and increases the risk of systemic side effects [132, 133].

**Fig. 4:**
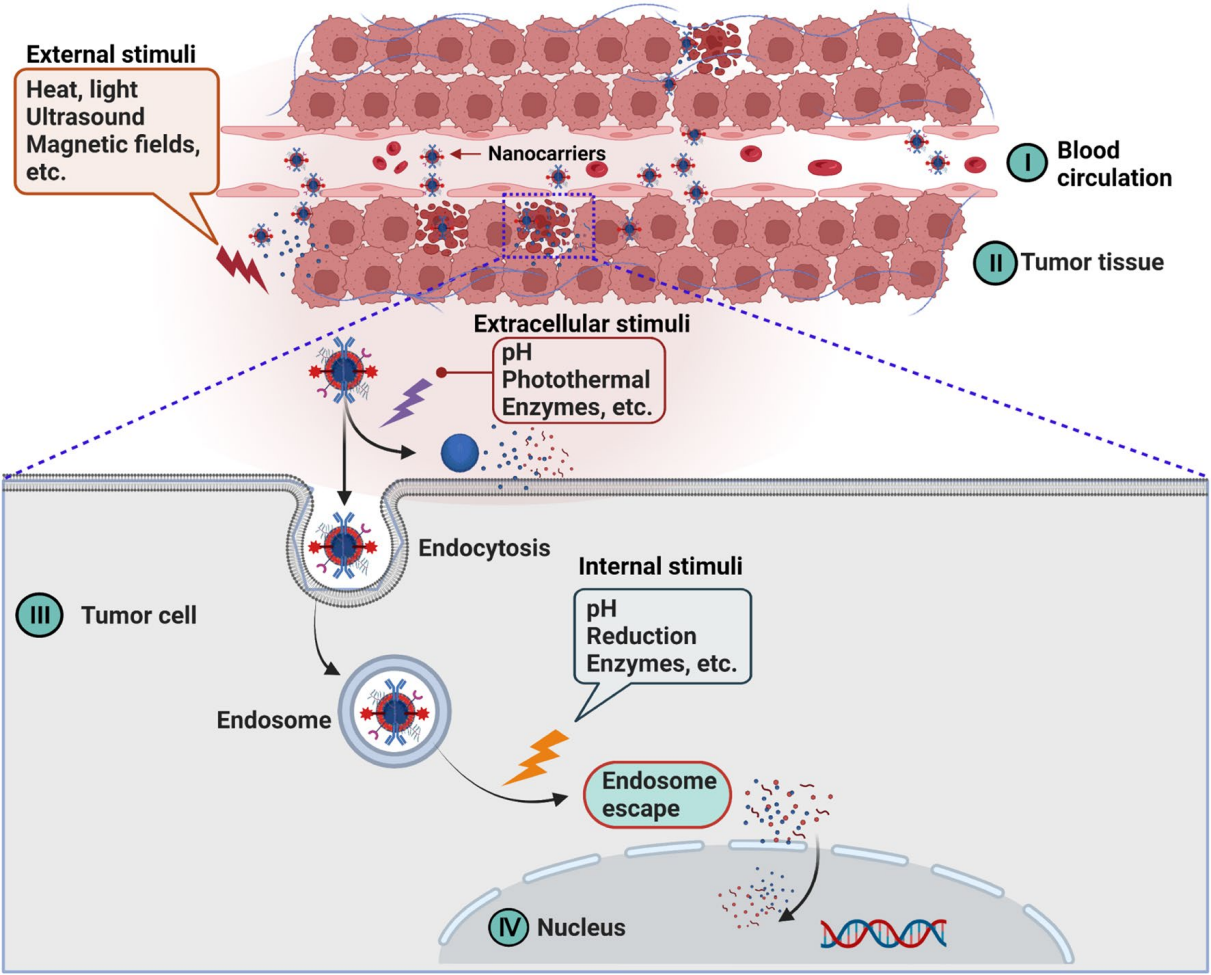
Nanocarriers penetrate from blood circulation (I) into the tumor nucleus (IV) and are responsive to different stimuli. (I − II) Drug delivery systems based on nanocarriers can enhance drug penetration and retention into the tumor tissues via the endothelial gap. Externally stimuli (e.g., heat, light, ultrasound, magnetic fields, etc.) or extracellular stimuli (e.g., acidic TME, thermal radiation, enzymes, etc.) can cause the nanocarriers to release their cargos in the vicinity of the target tissue. (III − IV) Intracellular nanocarriers escape from the endosomes and release their drugs in response to internal stimuli (e.g., pH, reduction, enzyme, etc.) and specifically bind to the target to exert anticancer effects. The figure has been created using Biorender

Controlled delivery systems, which are used to load chemotherapeutic agents and provide sustained release, will address these limitations of the lipiodol-TACE system and, hopefully, increase patient comfort and compliance [134]. A more precise dose of chemotherapy can be delivered to the tumor tissue as the chemotherapeutic agents loaded into the drug-delivery carriers can be controlled. The exploration of stimulus-responsive nanocarriers has been devoted to great efforts recently. As shown in Fig. 4, controlled delivery nanocarriers are accompanied by triggered drug release in response to either extracellular stimuli (pH, photothermal, enzymes) or intracellular stimuli (pH, reduction, and enzymes) [45]. In addition, some external stimuli such as heat, light, ultrasound, and magnetic fields also initiate the primary release of nanocarriers. Among many stimuli, pH sensitivity is most exploited to trigger drug release [135].

In general, the TME is weakly acidic (pH 6.8 − 7.2), with a pH of 5 − 6 in the endosomes and a pH of 4.5 − 5.5 in the lysosomes [136]. Such an acidic pH gradient guarantees the pH-responsive release of drugs loaded on nanocarriers. Liu et al. developed a pH-responsible magnetic DOX-loaded nano-system mixed with lipiodol for MRI-guided photothermal-chemoembolization treatment in an orthotopic liver tumor model [74]. This nano-system exhibited unambiguous pH-triggered drug release properties at acid TME because of the introduction of the pH-sensitive dopamine (PDA). Such pH-dependent control release behavior can be attributed to promoting protonation of hydroxyl in PDA molecules with reduced pH value, which weakens the electrostatic bond between PDA and DOX molecules [74]. Another research reported that a multifunctional nano-system (UiO-66/Bi_2_S_3_@DOX) could simultaneously achieve photothermal effects and low pH-triggered DOX release. In this study, DOX release behavior was regulated by both pH and NIR laser, which benefited the combined chemical and photothermal therapy. The nano-system exhibited a pH-responsive release behavior and an excellent photothermal effect in a series of in vitro and in vivo studies [75]. In addition, Lym et al. developed a novel sulfamethazine-based anionic pH-sensitive block copolymer (PCL-PEG-SM) as an alternative approach for TACE [69]. The copolymer underwent a sol-to-gel phase transition upon altering the environmental pH. The release of DOX from DOX-loaded copolymer hydrogels could be sustained for more than four weeks in vitro, and the released DOX retained its full bioactivity via inhibition of the proliferation of hepatic cancer cells [69]. Undoubtedly, these studies demonstrate that these pH-responsive drug delivery platforms may be promising therapeutic agents for enhancing TACE therapy for HCC treatment.

### Functionalized embolic agent

Embolization is one of the essential steps in the TACE procedure. Multifunctional nanoplatforms have been developed as an embolization system to facilitate transcatheter embolization. At present, there are many materials used for vascular embolization [137]. Usually, the embolic agents used for TAE of HCC in clinical practice include granular embolic agents represented by microspheres and liquid embolic agents represented by lipiodol [12, 14]. These embolic agents enter the tumor blood supply artery through the hepatic artery via the catheter to achieve embolization by blocking the tumor’s blood supply. Like these agents, multifunctional nanoplatforms can also be developed as solid embolization systems (e.g., microsphere complexes) or fluid embolization systems (e.g., hydrogel) to achieve embolization.

#### Solid embolization system

**Fig. 5:**
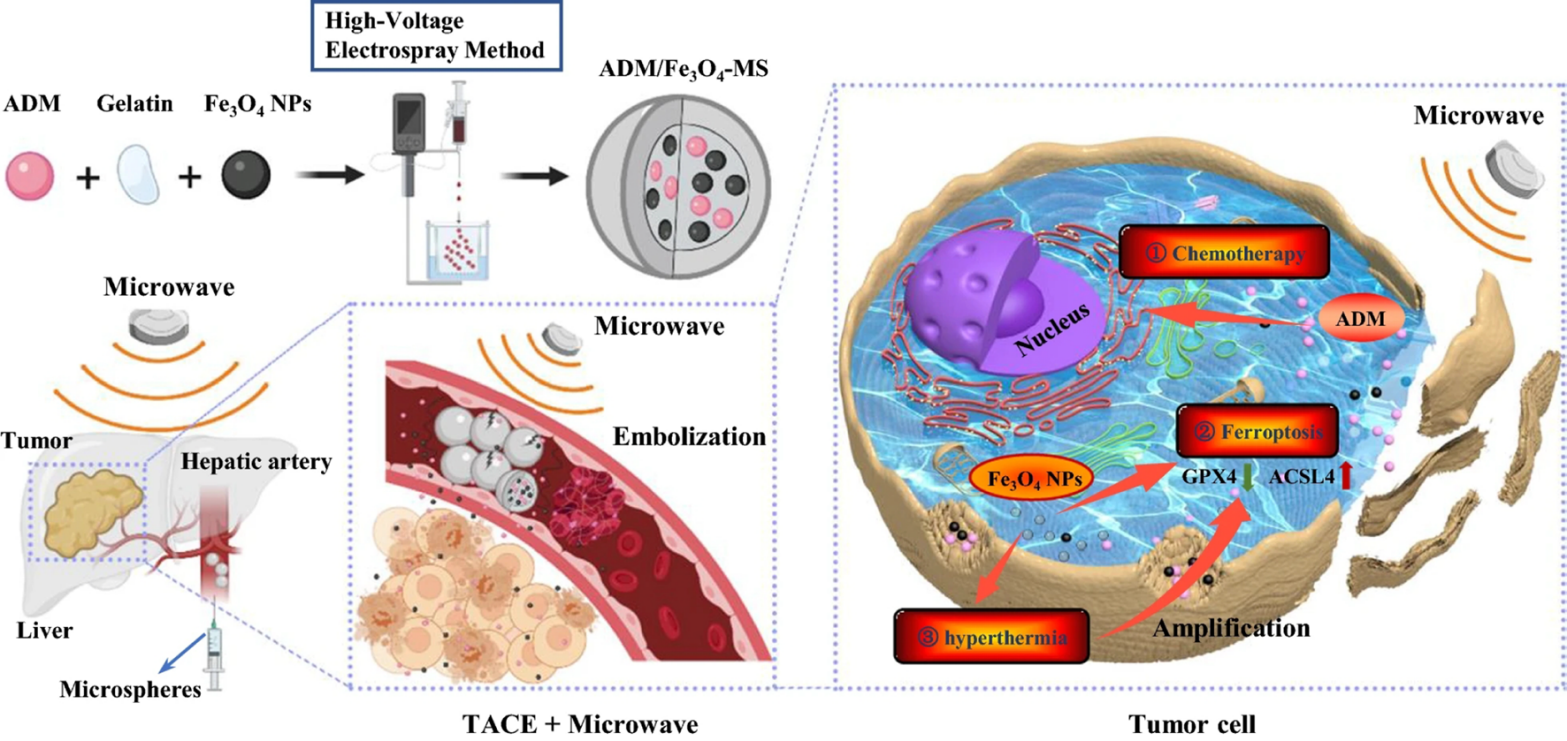
Schematic diagram of the preparation process of ADM/Fe_3_O_4_-MS and its TACE treatment mode. Reproduced with permission from Chen et al. [60]. Copyright 2022, BMC

As a commonly used solid embolic agent of TACE in clinical practice, microspheres are widely used in treating HCC [14]. Increasing evidence shows that drug-eluting microspheres have obvious advantages in reducing systemic side effects of chemotherapy drugs [26, 138]. The multifunctional nanoplatform developed by incorporating nanoparticles into microspheres can be used as an embolic agent to promote transcatheter embolization. As shown in Fig. 5, a multifunctional composite microsphere (ADM/Fe_3_O_4_-MS) is synthesized by incorporating ADM and Fe_3_O_4_ NPs into gelatin with a high-voltage electrospray technology [60]. In addition to having an excellent embolization effect, it can disintegrate and release the drug ADM and Fe_3_O_4_ NPs under microwave stimulation in vitro. Fe_3_O_4_ NPs can provide excellent T2-weighted MRI performance and significantly enhance the anticancer effect via ferroptosis under microwave-induced hyperthermia. In vitro and in vivo studies showed that the multifunctional embolization system had significantly better anti-tumor efficacy than other microspheres. To promote transcatheter embolization, another research developed a biodegradable multifunctional porous microsphere (BMPM) composed of carrageenan (Additional file 1: Fig. S3), whose good roundness and excellent swelling behavior guarantee them to be smoothly transported into targeted arteries’ ends and achieve satisfactory embolization effect [53].

Although the microspheres as embolic agents have many advantages in TACE application, it is challenging to deliver the anti-cancer drug into tumor tissue for the micron-sized embolic microspheres due to the physiological barriers posed by the remarkably abnormal TME to medication delivery. To solve these problems, Li et al. developed a novel multifunctional embolic microsphere with micro-nano binary progressive structure (MN-Ms) for TACE applications [48]. As expected, a better anti-tumor effect for MN-Ms was obtained by embolizing the tumor-feeding arterial vessels, delivering chemotherapeutic drugs to the targeted tumor tissue, and inhibiting the reestablishment of collateral circulation.

#### Fluid embolization system

Currently, liquid embolic agents are at the forefront of chemoembolization development because they allow deeper penetration of tumor blood vessels, increase tumor exposure to therapeutic drugs, and prevent revascularization by occupying the vasculature of various diameters, which is different from solid embolic particles [24]. Lipiodol is the first liquid embolic agent used in TACE therapy, with a history of decades. Lipiodol-based c-TACE is a common approach for treating middle and advanced HCC [11]. There are two other liquid embolic agents on the market, Onyx® (Medtronic plc) and PHIL™ (MicroVention, Inc.), which are mainly applied to embolize intracranial and peripheral arteriovenous malformations (AVMs) [139, 140]. As these embolic agents often need to be dissolved in DMSO for clinical application, it is risky that they may induce vascular inflammation and angionecrosis [141]. Moreover, neither Onyx nor PHIL can be used to deliver drugs as the dissipation of DMSO during administration would cause a burst release of the entire therapeutic payload, resulting in acute local toxicity and transient therapeutic effects. Therefore, these embolic agents cannot be used as chemoembolic agents for TACE [24].

**Fig. 6:**
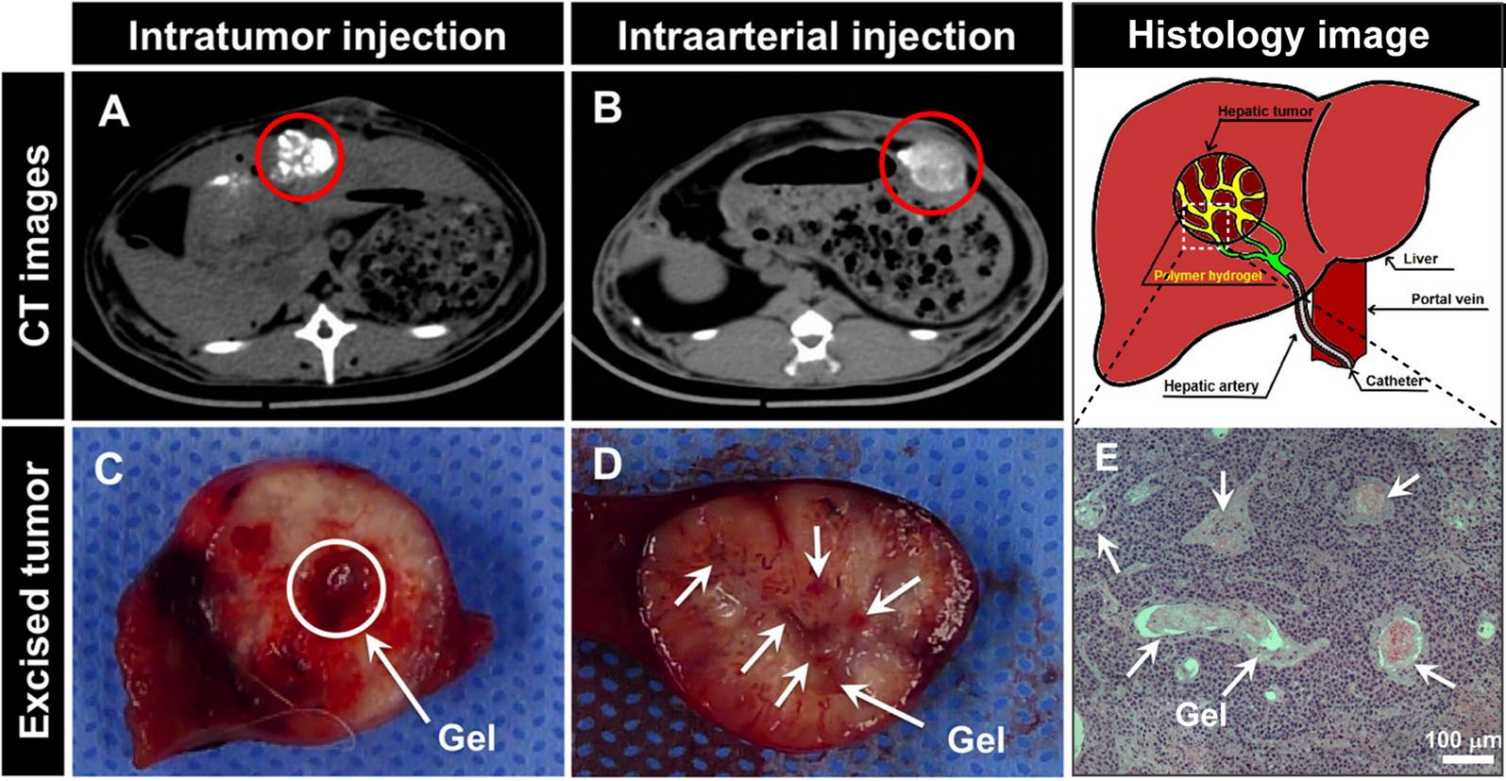
The in vivo gelation ability of the radiopaque embolic solutions, which contain copolymer (25 wt%), lipiodol^®^ (10 wt%) and DOX (10 mg mL^−1^), was observed at 5 h after injection. The white color of the radiopaque signals in red circles on CT images (**A**, **B**) and the red regions in white circles or indicated arrows in the excised tumors images (**C**, **D**) indicated the gelation of the radiopaque embolic materials after intratumoral (**A**, **C**) or intraarterial (**B**, **D**) injection. **E** H&E-stained histology image of excised tumor at 5 h after intraarterial injection in which the gel regions were indicated by arrows. Reprinted with permission from Lym et al. [69]. Copyright 2016, Elsevier

Although no other liquid embolization agent for TACE except for lipiodol has been developed in clinical, some drug-loaded liquid embolization systems, which can be applied through intratumor or TACE-based intraarterial injection, have been studied in vitro and in vivo with considerable breakthroughs [23, 68, 69]. As shown in Fig. 6, Lym et al. developed a novel sulfamethazine-based hydrogel, an anionic pH-sensitive block copolymer named PCL-PEG-SM, with potential application as a radiopaque embolic material [69]. Unlike lipiodol, this liquid embolization agent can not only be injected directly intratumor or perfused intraarterial after loading chemotherapy drugs but also show a remarkable sol–gel phase transformation to form a solidified state once delivered to the tumor site, thus effectively blocking the nutrient supply to tumor tissue (Fig. 6). In addition, incorporating iodine-containing contrast agents into the delivery system can also achieve real-time imaging monitoring during tumor treatment and postoperative imaging follow-up of the tumor lesions.

Undoubtedly, these studies show that the multifunctional nanoplatform plays a positive role in improving the embolization efficiency of tumor vessels as an embolization system.

### Synergistic therapies

#### Chemo-photothermal therapy

**Fig. 7:**
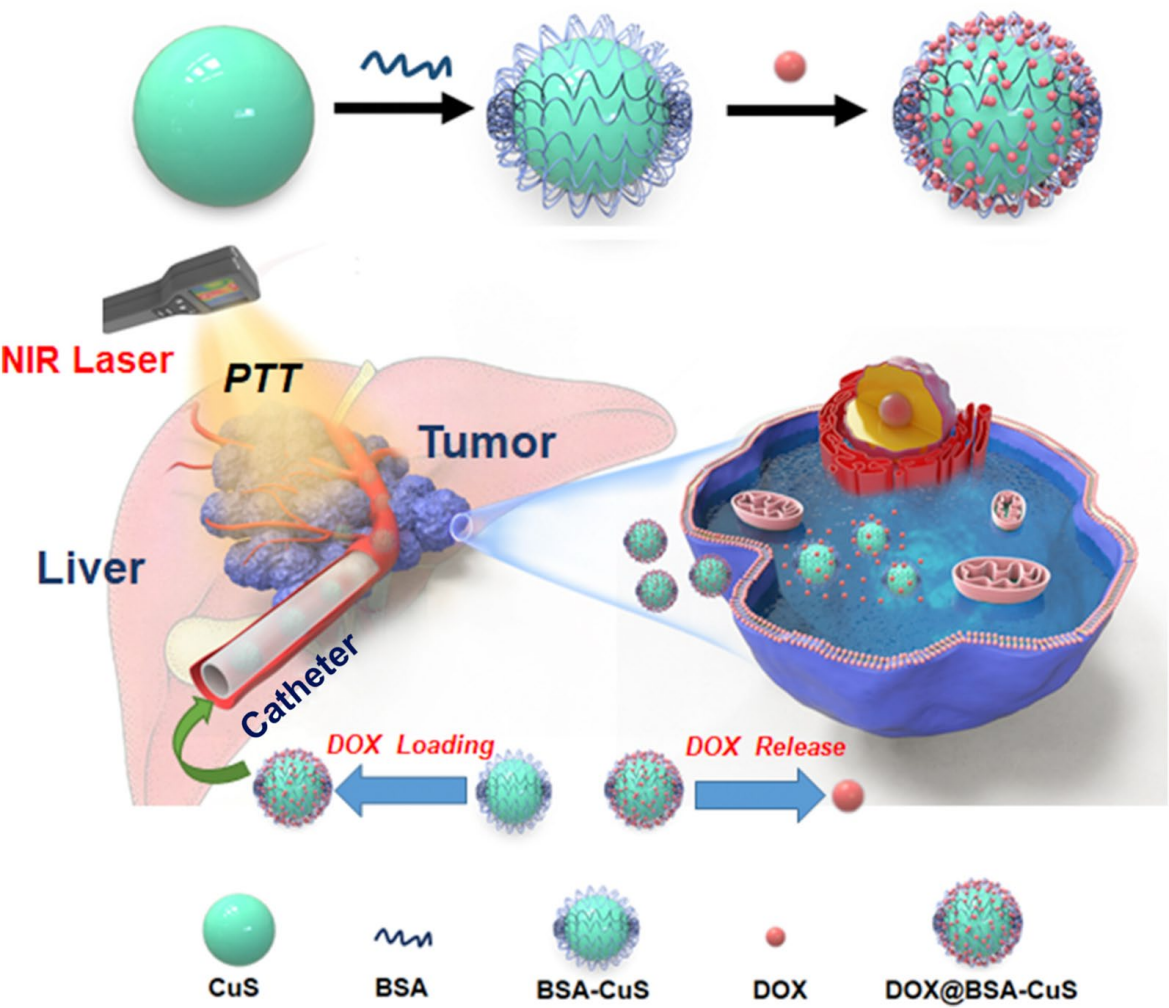
Schematic illustration of DOX@BSA-CuS NP preparation and synergistic therapy of orthotopic liver cancer by chemo-photothermal treatment through an intra-arterial intervention administration process. Reprinted with permission from Li et al. [76]. Copyright 2021, Elsevier

Ablation, such as radiofrequency or microwave ablation, is a minimally invasive treatment based on thermal effects, which has been clinically recognized as a radical method for HCC [1]. However, these methods can only be applied to the early stage of HCC [142]. Nanomaterials with thermal effects are not limited by tumor stage in the application, and a hybrid embolization system can be developed to satisfy both TACE therapy and thermal ablation to maximize the treatment of tumor tissue. As a synergistic therapeutic strategy, chemo-photothermal systems based on nanomaterials have shown unique advantages in HCC. Figure 7 illustrates the preparation of a drug-loaded chemo-photothermal system and synergistic therapy mechanism for HCC. The DOX-encapsulated and near-infrared (NIR)-responsible copper sulfide (CuS)-based (DOX@BSA-CuS) nanotherapeutics were developed for MRI-guided chemo-photothermal therapy of orthotopic HCC tumors in rats [76]. The as-prepared BSA-CuS NPs exhibited more substantial absorbance in the NIR region and photothermal effect to kill cancer cells efficiently. Besides, the external NIR laser-induced heating triggered the release of DOX synchronously. Furthermore, the DOX@BSA-CuS and lipiodol were transarterially perfused through a catheter to simultaneously deliver chemotherapy and phototherapy locally and ablate the tumor, synergistically playing the optimal treatment effect (Fig. 7).

With the development of nanotechnology, more and more chemo-photothermal systems such as DOX-loaded α-Fe_2_O_3_@PDA [74], DOX-loaded SPION@PDA [54], DOX@HAuNS [63], and UiO-66/Bi_2_S_3_@DOX [75] have been developed for the treatment of HCC. In addition, magnetic nanoparticles (MNPs) have been developed for chemo-photothermal therapy owing to their unique magnetic hyperthermia [60, 143]. A recent study designed a novel magnetic drug carrier system consisting of micron iron powder, barium ferrite (BaFe_12_O_19_) and carbon-coated iron nanocrystals (CCIN). After magnetization, BaFe_12_O_19_ can simultaneously adsorb CCIN and iron powder to form large clusters. Under the effect of the medium-frequency magnetic induction device, the magnetic induction temperature can reach 43 °C within 1 min, and the highest temperature can reach 70.8 °C within 2.5 h [144]. These multifunctional NPs carrier systems can simultaneously achieve multiple therapeutic effects of enhanced chemotherapy, embolization, and thermal ablation, which are expected to become a new method for treating HCC.

#### Sensitizing lipiodol-TACE

Lipiodol is not only a classical embolization agent but also an ideal X-ray contrast agent. Although it faces the drawbacks of rapid drug release and unstable properties of lipiodol emulsions in c-TACE treatment process [145], it still plays a vital role in the chemoembolization of HCC.

Several approaches have been devoted to improving the stability of hydrophilic chemotherapeutic drugs in lipiodol [49, 66, 67, 146]. The combination of nanocarrier with lipiodol-TACE in treating HCC can synergistically enhance the therapeutic effect of c-TACE. Shen et al. developed a new type of “oil-soluble” nanocarriers, named reversed lipid-based NPs (RLBNs), for the delivery of DOX, which was dispersed in lipiodol for TACE treatment of rat liver cancer. Compared with c-TACE system, this nanocarrier embolization system possesses a hydrophobic nanostructure with a high dispersibility in oils, significantly sustained drug release characteristics, lower side effects, and significantly enhanced inhibition of tumor growth [49]. Another attractive approach to stabilizing lipiodol emulsion is to use Pickering technology to obtain biodegradable, remarkably stable water-in-oil (W/O) lipiodol emulsion by adding PLGA NPs into the aqueous-phase of the formulation and emulsifying the solution using two connected syringes [67]. Compared with usual emulsions stabilized with synthetic surfactants, this W/O Pickering emulsion stabilized with these NPs has the advantage of being biodegradable, biocompatible, and less toxic.

**Fig. 8:**
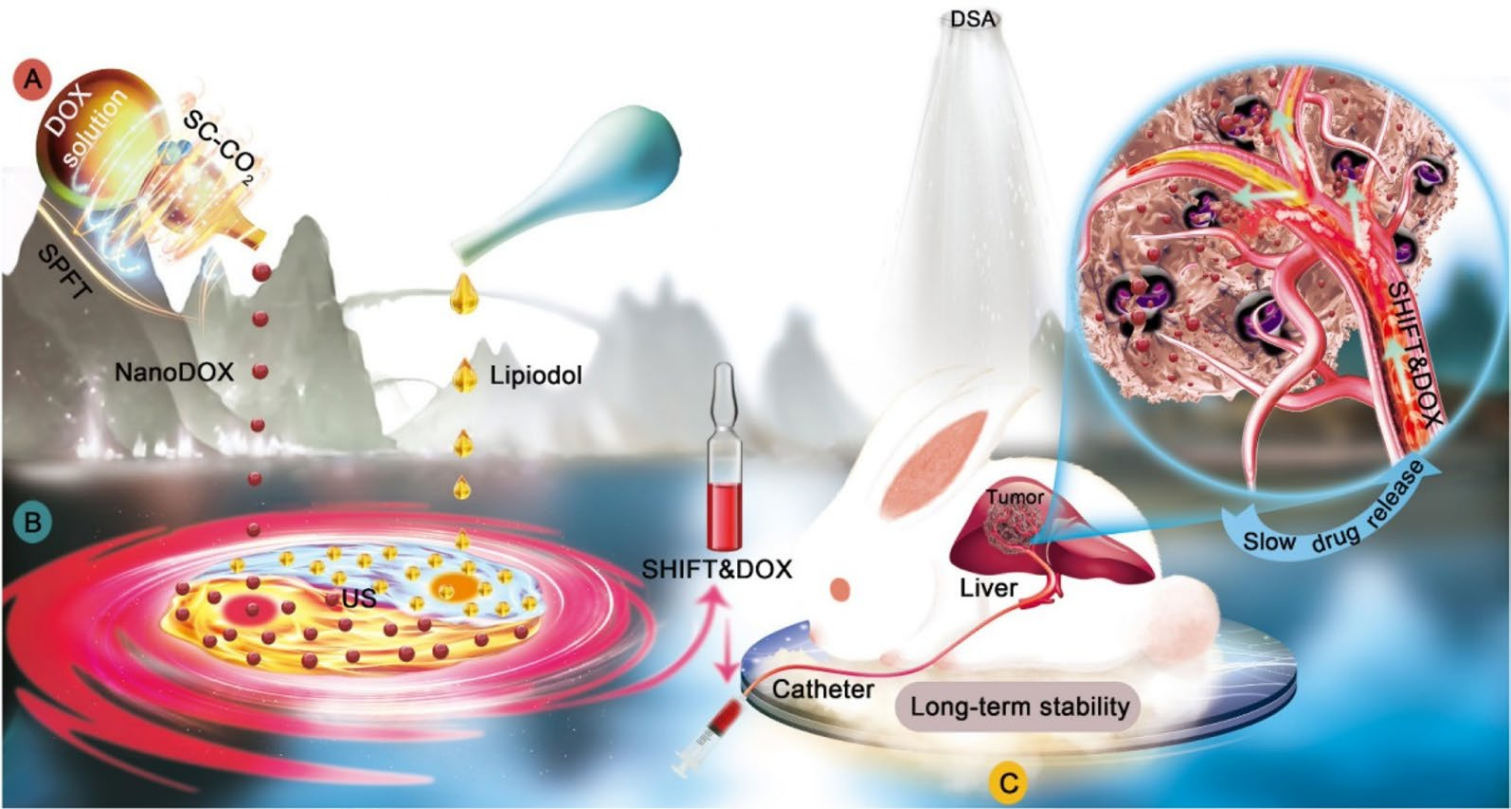
Schematic illustration of SHIFT&DOX preparation and transarterial chemoembolization of HCC. **A** Superstable pure-nanomedicine formulation technology (SPFT) was used to produce nano-DOX with a smaller nanoparticle size and homogeneity. **B** Subsequently, nano-DOX was homogeneously dispersed into lipiodol via ultrasonication (US) to prepare the SHIFT&DOX. **C** The SHIFT&DOX was specifically deposited into hepatocellular carcinoma lesions through transcatheter embolization. This led to long-term DOX stability and slow drug release from lipiodol to improve treatment effects and safety of HCC. Reproduced with permission from He et al. [146]. Copyright 2022, Ivyspring

Furthermore, a carrier-free hydrophilic drug nanotechnology-based super-stable homogeneous intermixed formulation (SHIFT) system was developed to overcome the lack of physical stability of TACE development [147, 148]. As shown in Fig. 8, the DOX solution was prepared as a pure nanodrug and then stably and homogeneously dispersed in lipiodol (SHIFT&DOX) by slightly ultrasonic dispersion. In vivo results showed that, unlike the conventional mixture of DOX and lipiodol, SHIFT&DOX exhibited excellent prolonged drug retention and sustained drug release effect in tumor lesions, which provided a promising approach to solving the instability of the traditional lipiodol emulsion and sensitizing the therapeutic effect of lipiodol-TACE in clinic [146].

## Research status of nanomaterial for TACE therapy in the treatment of HCC

### Clinical researches

Although ablation, surgery, and liver transplantation are radical treatments for HCC, the outlook suggests that new nano-conditioned therapies based on TACE will be helpful in clinical practice [72]. Researchers have set the goal of developing nanoplatforms to apply nanomaterials in clinics so as to improve the efficacy of TACE in treating HCC and reduce systemic side effects.

As early as 2006, Dudeck et al. sought to prove the feasibility of selective arterial infusion of SPIO particles in patients with HCC; all patients underwent MRI as a baseline and immediate follow-up investigation [50]. Although only 13 cases were included, this study laid the foundation for using SPIO NPs in the treatment of HCC by TACE. Recently, a study explored the value of TACE combined with a targeted NPs delivery system for sorafenib (SFB) to treat HCC with microvascular invasion [97]. 42 HCC patients with microvascular invasion after liver cancer surgery were divided into an experimental group (18 cases treated with combination therapy of TACE and Ab-SFB-NP system) and a control group (24 cases who took TACE and non-nano drug delivery system) based on their willingness. The results showed that these polymer NPs had high adoption value and high security, which could become a new nanomedicine platform for liver therapy, and patients with advanced HCC who cannot be treated with surgery were given some hope of survival. In addition, as a common nanocarrier, liposomes have also shown apparent advantages in the TACE delivery system. By encapsulating DOX within a liposome of polyethylene glycol (PEG), PEGylated liposomal DOX (PGLD) can reduce DOX toxicity and block the drug recognized by the mononuclear macrophage system, thus prolonging its circulation [149, 150]. Liao et al. conducted a clinical study which revealed that combined therapy with raltitrexed and PGLD in TACE could alleviate adverse chemotherapy responses in patients with HCC and improve overall survival (OS) [47]. Undoubtedly, although additional extensive, randomized controlled studies are needed to evaluate the clinical application value of these therapeutic approaches, the results presented in these clinical studies have already provided a promising palliative therapeutic option for patients with unresectable HCC.

### Basic experimental research

At present, although some single-center and small-scale clinical studies have attempted to apply nanoplatforms in the treatment of HCC by TACE and have achieved encouraging results, it is worth noting that most of the studies on the application of multifunctional nanoplatforms in TACE against HCC are still at the basic stage. As shown in Table 4, animal models (such as rats, rabbits, or swine) are still the main research subjects of nanoplatforms for the diagnosis and treatment of liver cancer. As one of the most used animal models, rats are cheap in price, easy to obtain, and relatively easy to model, so they are widely used in studying nanoplatform imaging diagnosis, drug delivery, and PTT [52, 56, 61, 98]. Whereas, due to the requirements of vascular size for interventional intubation, the study of rat liver cancer models for TACE is relatively rare. Currently, VX2 tumor-bearing rabbits remain excellent animal models for studying TACE therapy for HCC. Although the VX2 tumor is not hepatogenic, the rabbit VX2 liver tumor model has similar pathological and anatomic characteristics with HCC patients, especially since the tumor's blood supply is very similar to that of HCC. Thus, this model is a classic animal model for interventional embolization of HCC, which plays a crucial role in studying the application of multifunctional nanoplatforms in TACE therapy of HCC [39, 58, 65, 78, 151–153]. In addition, apart from the above two animal models, the swine model is also considered suitable for TACE study as it has the advantages of high homology to humans and is suitable for interventional intubation. However, it has not yet been widely used since the high cost and complexity of modeling, and only a few studies have conducted feasibility demonstration studies on transarterial intubation, imaging, and pharmacokinetics in healthy porcine models [36, 154, 155].

**Table 4:** Different models for the study of TACE treating HCC with nano-formulation

Species	Models	Characteristic	Research field	Refs.
Human	HCC patients	Clinical trials, high level of evidence	Image (MRI); drug delivery	[47, 50, 51, 97]
Rat	N1S1 liver tumor; Mca-rh7777 rat; ACI rat (Morris hepatoma 3924A)	Low cost, easy to obtain, breed and raise, relatively easy to modeling	Image (MRI); drug deliver; embolization; PTT, targeting therapy	[49, 52, 53, 56, 61, 74–76, 96, 98, 146]
Rabbit	VX2 liver cancer; rabbit ear embolization model	Similar pathological and anatomic characteristics with HCC patients, high reproductive, large body size, and suitable for TACE procedure	Image (US, X-ray, MRI); drug deliver; embolization; gene therapy; PTT; PDT; microwave ablation	[36, 54, 55, 57–60, 62–66, 68, 69, 78, 107, 113, 146, 156]
Swine	Porcine (healthy); PBPK model	High homology to human, suitable for interventional intubation	Embolization; image (X-ray); pharmacokinetic profiles; drug deliver	[36, 154, 155]

*PBPK* porcine semi-physiologically based pharmacokinetic

## Limitations

Obviously, numerous studies have shown that applying multifunctional nanoplatforms in TACE therapy for HCC has made great progress. However, some bottleneck problems restricting the efficacy of TACE still need to be further solved. (1) While multifunctional nanoplatforms endow nanomaterials with multiple functions, it inevitably needs to incorporate more substances, and the material safety issue brought by this is still a huge challenge for researchers. (2) A series of changes in TME after TACE, such as accumulation of acid metabolic products, hypoxia-induced angiogenesis, resistance, and autophagy activation, restrict the efficacy of TACE therapy. Therefore, how to deal with the changes in these microenvironments after tumor embolization is still the focus of researches and it encourages researchers to develop new nanoplatforms. (3) Imaging follow-up after tumor embolization is essential for evaluating the therapeutic effect. At present, there is still a lack of stable and effective imaging follow-up nanoplatforms; therefore, developing multifunctional nanoplatforms capable of image follow-up for TACE therapy is urgent. In short, there is still a long way to go for multifunctional nanoplatforms from basic research to clinical application, and only continuous innovation can achieve breakthroughs.

## Conclusions and perspectives

Recent studies on the improvement of TACE nanomaterials are very promising. Based on the review of previous relevant studies, we believe that the most significant advantage of multifunctional nanoplatforms in TACE treatment of HCC is that it can not only improve the distribution of chemotherapy drugs in local tumor tissues but also avoid the serious side effects caused by systemic drug administration. Moreover, with special physical and chemical properties, the nanoplatforms can be combined with TACE to play a synergistic anticancer role and enhance the therapeutic effect. The minimally invasive interventional technique dramatically improves nanocarriers' tumor targetability and antitumor efficacy. Improvements in the therapeutic effect of TACE may be possible via the development of new multifunctional nanoplatforms with properties such as detectability with imaging, targetability, efficient delivery and embolization, biodegradability, and the incorporation of multiple therapeutics or multiple therapeutic modalities. Besides, more choices are available for applying new nanoplatforms in TACE for the precise therapy of HCC with rapid development of nanomaterials and significant progress in nanotechnology. However, most studies of multifunctional nanoplatform were only carried out in animal models and have not yet been applied to large-scale in vivo studies in patients. Therefore, more efforts such as preclinical studies of the biosafety and anticancer efficacy of nanoformulations as drug carriers are still eagerly needed to pave the way for the translation of new nanoformulations into clinical use and thus to improve the overall patient outcome of HCC treatment. On the other hand, nanotechnology and transcatheter arterial embolization techniques in animals do not seem to fully realize their potential so far. The underlying applications in each field still need to be developed.

In conclusion, although the multifunctional nanoplatforms have not yet been widely used in clinical practice, and some thorny issues that limit the efficacy of TACE also need to be solved urgently, these studies have undoubtedly provided a broader vision for clinical TACE treatment of HCC. In addition, the potential of some emerging cancer treatments based on nanomedicine, such as chemodynamic therapy (CDT) [157], molecular dynamic therapy (MDT) [158], and immunotherapy [159], provides more new opportunities and challenges for researchers to develop some innovative multifunctional nanoplatforms to improve the efficacy of TACE in the treatment of HCC. The design concepts, functional characteristics, and therapeutic effects of these nanoplatforms provided reference for developing more appropriate and effective embolization materials. Therefore, based on these previous studies, it is highly expected that some novel multifunctional nanoplatforms with higher efficacy and safety will be developed and applied in the TACE therapy of HCC.

## Supplementary Information

**Additional file 1**: **Fig. S1** Schematic illustration showing the use of newly developed DOX-NPs-MB complex in lipiodol formulation to enhance drug delivery via ultrasound irradiation (US+) during TACE procedure. Reproduced with permission from Kim et al [1]. Copyright 2021, Ivyspring. **Fig. S2 **Schematic illustration of drug loading, cellular entry, drug release, and antitumor mechanism of LHD/miR-375. Reproduced with permission from Xue et al [2]. Copyright 2017, Dove Medical Press. **Fig. S3** A brief introduction about BMPMs’ ingredients, structure as well as working mechanism on iTACE. Reproduced with permission from Liu et al [3]. Copyright 2020, Elsevier.

## Data Availability

Not applicable.

## Abbreviations

HCC: Hepatocellular carcinoma
TACE: Transarterial chemoembolization
BCLC: Barcelona clinic liver cancer
DEBs: Drug-eluting beads
DOX: Doxorubicin
NPs: Nanoparticles
FDA: Food and Drug Administration
SFB: Sorafenib
ECM: Extracellular matrix
P-gp: P-glycoprotein
HA: Hyaluronic acid
HACE: Hyaluronic Acid-ceramide (HACE)
VEGF: Vascular endothelial growth factor
EPR: Enhanced permeability and retention
PAH: Polyallylamine hydrochloride
PAA: Polyacrylic acid
CPPs: Cell-penetrating Peptides
TME: Tumor microenvironment
CCIN: Carbon-coated iron nanocrystals
RLBNs: Reversed Lipid-based NPs (RLBNs)
SHIFT: Super-stable homogeneous intermixed formulation
