# Supplementary material for: Multifunctional nanoplatforms application in the transcatheter chemoembolization against hepatocellular carcinoma
Source: J Nanobiotechnology. 2023 Feb 27;21:68. doi: 10.1186/s12951-023-01820-7 (PMC9969656; doi:10.1186/s12951-023-01820-7)
Supplement: Supplementary file 1 — Additional file 1: Fig. S1 Schematic illustration showing the use of newly developed DOX-NPs-MB complex in lipiodol formulation to enhance drug delivery via ultrasound irradiation (US+) during TACE procedure. Reproduced with permission from Kim et al [1]. Copyright 2021, Ivyspring. Fig. S2 Schematic illustration of drug loading, cellular entry, drug release, and antitumor mechanism of LHD/miR-375. Reproduced with permission from Xue et al [2]. Copyright 2017, Dove Medical Press. Fig. S3 A brief introduction about BMPMs’ ingredients, structure as well as working mechanism on iTACE. Reproduced with permission from Liu et al [3]. Copyright 2020, Elsevier. [file 12951_2023_1820_MOESM1_ESM.docx]

Additional file Information

**Multifunctional Nanoplatforms Application in the Transcatheter Chemoembolization against Hepatocellular Carcinoma**

Gang Yuan^1,2#^, Zhiyin Liu^3#^, Weiming Wang^2,4#^, Mengnan Liu^2,5^, Yanneng Xu^1,2^, Wei Hu^1,2^, Yao Fan^6^, Xun Zhang^1^, Yong Liu^4^*^*^*, Guangyan Si^1^*^*^*

1. Department of Intervention Radiology, Traditional Chinese Medicine Hospital Affiliated to Southwest Medical University, Luzhou 646000, China

2. State Key Laboratory of Quality Research in Chinese Medicine, Macau Institute for Applied Research in Medicine and Health, Macau University of Science and Technology, Taipa, Macau SAR, China

3. Department of Neurology, The Affiliated Hospital of Southwest Medical University, Luzhou 646000, China

4. Department of General Surgery (Vascular Surgery), The Affiliated Hospital of Southwest Medical University, Luzhou 646000, China

5. National Traditional Chinese Medicine Clinical Research Base and Department of Cardiovascular Medicine, The Affiliated Traditional Chinese Medicine Hospital of Southwest Medical University, Luzhou, China

6. Department of Anus and Intestine Surgery, Traditional Chinese Medicine Hospital Affiliated to Southwest Medical University, Luzhou 646000, China

*^*^*Correspondence should be addressed to Yong Liu (E-mail: lyong74@163.com), and Guangyan Si (Siguangyan@swmu.edu.cn)

^#^ Equal contributors and co-first authors.

**Supplementary figures**


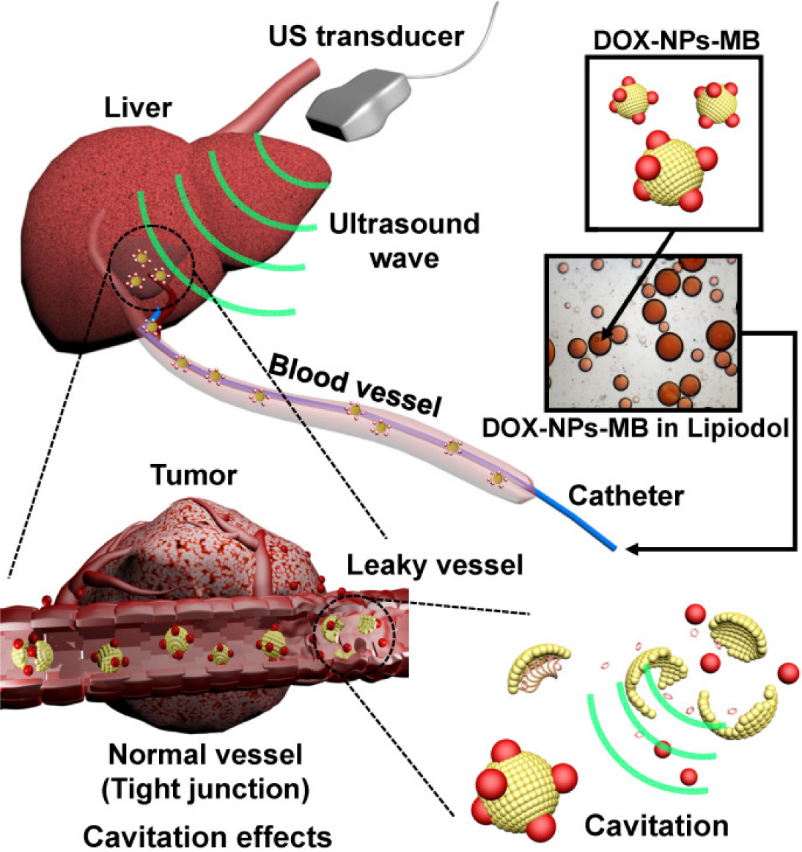


**Fig. S1** Schematic illustration showing the use of newly developed DOX-NPs-MB complex in lipiodol formulation to enhance drug delivery *via* ultrasound irradiation (US+) during TACE procedure. Reproduced with permission from Kim et al [1]. Copyright 2021, Ivyspring.


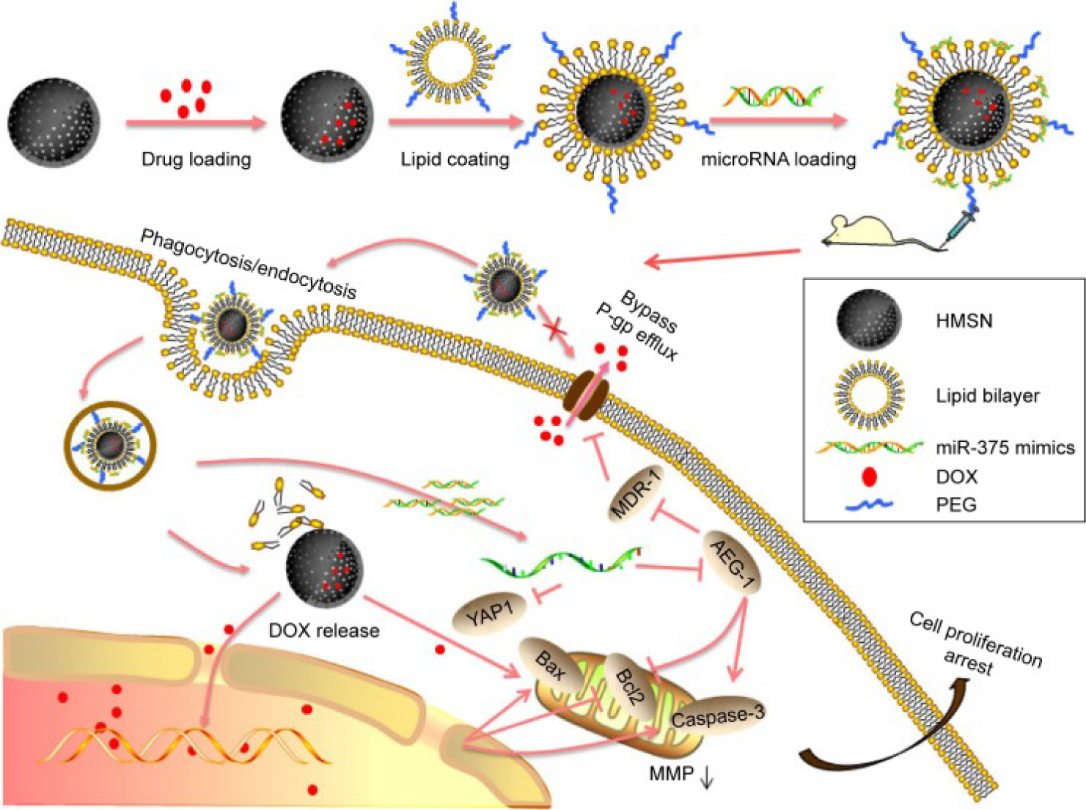


**Fig. S2** Schematic illustration of drug loading, cellular entry, drug release, and antitumor mechanism of LHD/miR-375. Reproduced with permission from Xue et al [2]. Copyright 2017, Dove Medical Press.


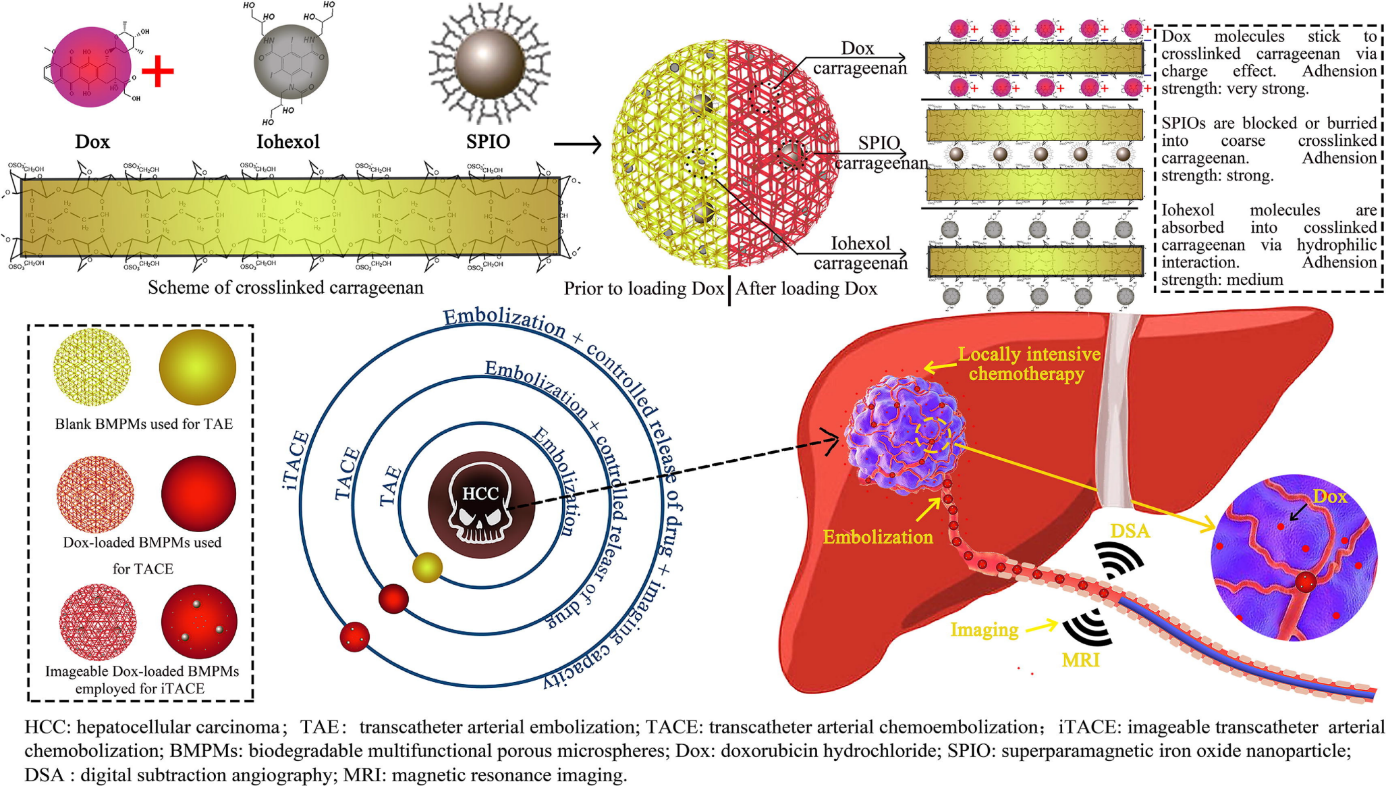


**Fig. S3** A brief introduction about BMPMs' ingredients, structure as well as working mechanism on iTACE. Reproduced with permission from Liu et al [3]. Copyright 2020, Elsevier.

References

[1] Kim D, Lee JH, Moon H, Seo M, Han H, Yoo H, et al. Development and evaluation of an ultrasound-triggered microbubble combined transarterial chemoembolization (TACE) formulation on rabbit VX2 liver cancer model. Theranostics. 2021; 11(1): 79−92.

[2] Xue H, Yu Z, Liu Y, Yuan W, Yang T, You J, et al. Delivery of miR-375 and doxorubicin hydrochloride by lipid-coated hollow mesoporous silica nanoparticles to overcome multiple drug resistance in hepatocellular carcinoma. Int J Nanomedicine. 2017; 12: 5271−87.

[3] Kunliang L, Zhicheng J, Xiaolong H, Dan Y, Yu Z, Haidong Z, et al. A biodegradable multifunctional porous microsphere composed of carrageenan for promoting imageable trans-arterial chemoembolization. Int J Biol Macromol. 2020; 142: 866−78.
